# Trainee anaesthetist self-assessment using an entrustment scale in workplace-based assessment

**DOI:** 10.1177/0310057X241234676

**Published:** 2024-04-22

**Authors:** Damian J Castanelli, Jennifer B Woods, Anusha R Chander, Jennifer M Weller

**Affiliations:** 1 School of Clinical Sciences at Monash Health, Monash University, Clayton, Australia; 2 Department of Anaesthesia and Perioperative Medicine, 2538 Monash Health, Clayton, Australia; 3 Department of Anaesthesia, 63588 Canterbury District Health Board, Christchurch, New Zealand; 4 Centre for Medical and Health Sciences Education, University of Auckland, Auckland, New Zealand

**Keywords:** Assessment, competence, education, educational measurement, e-portfolios, self-assessment

## Abstract

The role of self-assessment in workplace-based assessment remains contested. However, anaesthesia trainees need to learn to judge the quality of their own work. Entrustment scales have facilitated a shared understanding of performance standards among supervisors by aligning assessment ratings with everyday clinical supervisory decisions. We hypothesised that if the entrustment scale similarly helped trainees in their self-assessment, there would be substantial agreement between supervisor and trainee ratings. We collected separate mini-clinical evaluation exercises forms from 113 anaesthesia trainee-supervisor pairs from three hospitals in Australia and New Zealand. We calculated the agreement between trainee and supervisor ratings using Pearson and intraclass correlation coefficients. We also tested for associations with demographic variables and examined narrative comments for factors influencing rating. We found ratings agreed in 32% of cases, with 66% of trainee ratings within one point of the supervisor rating on a nine-point scale. The correlation between trainee and supervisor ratings was 0.71, and the degree of agreement measured by the intraclass correlation coefficient was 0.67. With higher supervisor ratings, trainee ratings better correlated with supervisor ratings. We found no strong association with demographic variables. Possible explanations of divergent ratings included one party being unaware of a vital aspect of the performance and different interpretations of the prospective nature of the scale. The substantial concordance between trainee and supervisor ratings supports the contention that the entrustment scale helped produce a shared understanding of the desired performance standard. Discussion between trainees and supervisors on the reasoning underlying their respective judgements would provide further opportunities to enhance this shared understanding.

## Introduction

Making accurate self-assessments of performance is essential to self-regulated learning and integral to lifelong learning and professional development.^1,2^ However, self-assessment represents something of a conundrum. Several authors have reported that self-assessment alone has a limited effect in producing change and improving performance because it correlates poorly with the judgements of others.^2,3^

In anaesthesia training, the ability of trainees to judge the amount of supervision they need for a case is critical to safe patient care. However, this ability cannot be taken for granted. For instance, in several medical settings, there is evidence that trainees want—or believe they require—less supervision than their supervisors think is necessary.^4–6^ Uncertainty in self-assessment may also contribute to the reported reluctance of trainees to ask for supervisory input in situations in which they believe supervisors expect them to be able to manage without direct supervision.^7^ Anaesthesia trainees must learn to judge where they are at, and where they are expected to be, so they may obtain the appropriate level of supervision for learning and safe patient care. Furthermore, they must learn how to regulate their own learning as independent practitioners after commencing specialist practice.

Part of the contention around self-assessment is that it has many meanings.^8–11^ In this paper, we view self-assessment as the integration of ‘both external and internal data, standards, and resources to inform and make decisions about one’s performance’.^9^ It is essentially a learning strategy^10^ that involves evaluating how you learnt and what you learnt, and coming to a judgement of your competence in performing a task.^11^ Rather than an individual process of ‘personal, unguided reflection’, self-assessment is better thought of as a social phenomenon, in which peers and supervisors can provide information about performance and standards and help the learner to make sense of performance data and plan future improvements.^10^

We know little about how anaesthesia trainees learn to judge their own performance or how we might facilitate this learning. However, we know the processes involved in self-regulated learning must be learnt and developed.^12^ To help facilitate this learning, the Australian and New Zealand College of Anaesthetists (ANZCA) introduced trainee self-assessment into their workplace-based assessments (WBAs). These WBAs use an entrustment scale.^13^ Previous research has shown that entrustment scales can help supervisors make more reliable assessments and improve the detection of poor performance.^14,15^ This improved reliability arises as the language of entrustment helps align assessment with the everyday decision making of supervisors on how much independence to grant a trainee.

Trainees make complementary decisions on how much supervision they need. It is possible that the entrustment scale may similarly help trainees harness this everyday decision making to inform their self-assessment, for example, ‘Am I comfortable doing this case with the supervisor at home or do I need them in the operating room?’. In this way, the entrustment scale (i.e. how much supervision is required/desired) may serve to convey a shared understanding of the desired performance standard. If this is true, then there should be a substantial agreement between supervisor and trainee ratings. In addition, as well as providing an opportunity for supervisors to facilitate trainees’ reflection and planning for future performance improvements, comparing the trainee’s self-assessment rating with the supervisor’s rating ought to prompt further discussion on their performance and the desired standards.^12,16^ By examining the reasoning behind divergent ratings, we might glean general insights into how trainees and supervisors make performance judgements, and what trainees might learn from such discussions.

Therefore, in this study, we examined the concordance between trainees’ and supervisors’ ratings for the level of supervision required for managing a case, and explored why those ratings may differ. Our research questions were:

1. To what extent do trainees’ and supervisors’ ratings concur on the level of supervision required for a case using the entrustment scale?
2. If they do not concur, why do trainees’ and supervisors’ ratings differ?

## Methods

Ethical approval for this study was granted by the Monash Health Human Research Ethics Committee (approval number RES-18-0000-723L, 20 December 2018) and the University of Otago Ethics’ Committee (reference H19/005). We obtained written informed consent from all participants.

### Context

In Australia and New Zealand, postgraduate anaesthesia training is centrally administered by ANZCA, a professional college, rather than individual universities or health services. In 2019, there were approximately 1600 ANZCA trainees in 160 training hospitals. Trainees progress through four stages of training (introductory, basic, advanced, provisional fellow) in variable time depending on when requirements are met and competence demonstrated. The minimum lengths are 6 months, 18 months, 24 months, and 12 months, respectively. The minimum training period is 5 years. To enhance learning and collect information on trainee performance, ANZCA uses a suite of WBAs. These are the mini-clinical evaluation exercises (mini-CEx) and direct observations of procedural skills for observed performance, case-based discussions for unobserved performance, and multisource feedback for longitudinal feedback. Trainees select the cases and supervisors for their WBAs. After an observed performance or discussion, the trainee reflects on their performance and selects an entrustment level before the supervisor provides feedback and awards a level of entrustment. All WBA data are entered into a central database maintained by ANZCA. (Full details of the ANZCA training programme can be accessed at www.anzca.edu.au/education-training/anaesthesia-training-program under Education and Training.) The entrustment scale used by ANZCA is a nine-point scale with three broad subdivisions.^17^ The explanations for the entrustment levels in the scales used by supervisors and trainees are presented in Tables 1 and 2. The mini-CEx form asks supervisors and trainees to use these scales to answer the question: ‘What level of supervision does the trainee require for THIS case overall?’.

**Table 1.** Supervisor view of the levels of entrustment.

Trainee needs the assessor in the theatre suite:	
1.	Not comfortable leaving trainee unsupervised in theatre for any period of time.
2.	Comfortable to leave trainee to go on brief coffee break in theatre tearoom. Not happy for trainee to instigate changes in management in your absence.
3.	As in 2, but comfortable staying out of theatre for a bit longer, e.g. while eating your lunch. Trainee may instigate some new actions that you have discussed.
Trainee needs the assessor in the hospital:	
4.	Happy to leave the theatre block, but remain immediately available in the hospital. Feels the need to check in on the trainee at regular intervals.
5.	Happy to leave the theatre block but remain immediately available in the hospital, e.g. not take on another case themselves. Expect trainee to notify supervisor of any significant problem or event, e.g. persistent abnormal physiological parameter, major blood loss.
6.	As in 5 but expect trainee to manage most problems initially, and call you if their initial management doesn’t work.
Trainee could manage this procedure independently and does not require direct supervision:	
7.	Could potentially be off-site but would want to review the trainee’s management plan before they started the case.
8.	Supervisor off-site. Confident that trainee can make a good assessment and plan, but want to be notified that they are doing the case.
9.	Trainee could manage this case as a supervisor. Appropriate if they don’t contact supervisor. May have a collegial discussion on case.

**Table 2.** Trainee view of the levels of entrustment.

I need the assessor in the theatre suite:	
1.	I do not feel comfortable being unsupervised in theatre for any period of time.
2.	I feel comfortable for my supervisor to go on a brief coffee break in the theatre tearoom. I do not feel comfortable instigating changes in management with my supervisor absent.
3.	Same as 2, but comfortable with supervisor staying out of theatre for a bit longer, e.g. while eating lunch. I feel comfortable instigating some new actions that have been previously discussed with my supervisor.
I need the assessor in the hospital:	
4.	I am happy for my supervisor to leave the theatre block but remain immediately available in the hospital. My supervisor needs to check in with me at regular intervals.
5.	I am happy for my supervisor to leave the theatre block but remain immediately available in the hospital, e.g. not take on another case themselves. I must notify my supervisor of any significant problem or event, e.g. persistent abnormal physiological parameter, major blood loss.
6.	Same as 5, but I can manage most problems initially, but will call my supervisor if my initial management doesn’t work.
I could manage this procedure independently and do not require direct supervision:	
7.	My supervisor could potentially be off-site but I would want my supervisor to review my management plan before starting the case.
8.	My supervisor is off-site. I am confident I can make a good assessment and plan, but will notify my supervisor that I am doing the case.
9.	I could manage this case like a supervisor. Appropriate if I do not contact the supervisor. May have a collegial discussion on case.

### Study protocol

We prospectively collected data at two hospital sites in Australia and one in New Zealand. We invited all trainees and supervisors to participate. Volunteer trainee and supervisor pairs performed a mini-CEx assessment during their routine work. They prospectively completed separate paper versions of the ANZCA mini-CEx form and additional demographic questions (age, gender, experience level, times worked together). In addition to the entrustment rating, the mini-CEx form had two prompts to the supervisor for narrative feedback: ‘Examples of what was done well’ and ‘Areas that need improvement and action’. The trainee was asked for ‘reflection and comments’ and for an ‘action plan: based on my reflection and the feedback I have received I intend to …’. We added two supplementary questions for both supervisors and trainees to address our research question better:

- Please describe as precisely as possible the specific elements of this case that you felt required supervisory input—including distant supervisory input (less than a score of nine).
- Record an action plan to move the trainee/you to the next level of supervision, one step closer to independent practice.

While in practice trainees and supervisors may discuss the case before completing the assessment form and may even complete it together; a critical part of our analysis was the independence of the ratings and comments. We emphasised to trainees and supervisors that they needed to finalise their entrustment ratings and narrative comments separately prior to any discussion of the assessment. We used department education sessions, participant information forms, and data collection forms to reinforce this message.

To estimate a sample size prospectively, we postulated that a difference in mean ratings between supervisors and trainees of more than 0.5 would be meaningful for our primary outcome. We used an estimated standard deviation in ratings of two, which gave us a calculated sample size of 126 for a repeated measures t-test with *α* (two-tailed) = 0.05 and *β* = 0.2. We also planned to see if demographic features might explain some of any observed difference in ratings.

### Analysis

As our primary outcome measure, trainee entrustment in a case, generates two ratings (trainee and supervisor), we used a repeated measures t-test to determine if the observed difference between the mean ratings of trainees and supervisors was significantly different from zero. This measure gives us a comparison between trainee and supervisor ratings at the group level. We also used a Pearson correlation coefficient to examine the correlation between trainee and supervisor ratings, as in other studies of self-assessment.^18,19^ Correlation estimates the degree of association of two measures. However, even with a high correlation, the individual measures can still differ substantially.^20^ In this instance, while correlation is desirable, we are more interested in agreement, or the concordance between two measures of the one variable. To address this, we calculated the criterion-referenced intraclass correlation coefficient (ICC), which compares the variance between paired ratings with the total variance across all ratings.^21^ In this study, we used a two-way, random effects model for absolute agreement with single raters, which is known as ICC (2,1)^22^ or ICC (A,1),^23^ and assumes our raters and cases are chosen from a random sample of all cases and raters. As the ANZCA mini-CEx form divides the nine-point scale into three broad categories, the ICC was also re-calculated using these categories as a three-point scale.^24^

To identify possible predictors of differences in ratings (in either direction), we looked for correlations between rating difference and the collected demographic variables. As self-assessment ability parallels expertise development,^25^ we also looked at how the difference in rating varied with supervisor rating using Pearson correlation coefficient and chi-square testing. We used Microsoft Excel (Microsoft, Redmond, WA, USA) and IBM SPSS Statistics for Windows v27 (IBM Corp., Armonk, NY, USA) for statistical analysis.

We also reviewed the trainee and supervisor responses to our supplementary questions and the comments on the mini-CEx forms. We looked for concordance or discordance in the elements of the case requiring supervisory input and factors influencing rating. We especially focused on possible reasons for differences in entrustment ratings by analysing the supervisor–trainee pairs with greater rating discrepancies to look for similarities and differences in their underlying reasoning.

## Results

### Demographics

Although the Covid-19 pandemic limited recruitment, a total of 113 supervisor–trainee pairs participated in this study. There were 31, 30 and 52 pairs recruited at the three participating sites, with a response rate of 73%. Fifty-one per cent of trainees and 35% of supervisors were women, compared with 43% of trainees and 33% of ANZCA Fellows in Australia in 2020.^26^ Tables 3 and 4 present the distribution of experience among trainees and supervisors. Introductory and basic trainees made up only 27% of our sample, compared with 46% of Australian trainees,^26^ reflecting the higher proportion of advanced trainees at our recruitment sites. Table 5 presents the number of times trainees had worked with the supervisor prior to the mini-CEx. In 40% of assessments the trainee had worked with the supervisor more than five times.

**Table 3.** Trainee training level.

Level	Number	Percentage
Introductory training	5	4%
Basic training	26	23%
Advanced training	59	52%
Provisional fellow training	21	19%
Unrecorded	2	2%
Total	113	

**Table 4.** Supervisor experience.

Years	Number	Percentage
<5	42	37%
5–9	24	21%
10–14	18	16%
15–19	6	5%
20–24	9	8%
25 or more	12	11%
Unrecorded	2	2%
Total	113	

**Table 5.** Prior exposure of trainee to supervisor.

Times worked together	Number	Percentage
0	7	6%
1	10	9%
2	11	10%
3	18	16%
4	5	4%
5	17	15%
6–10	33	29%
11–20	12	11%
Total	113	

### Comparison of trainee and supervisor ratings

**Figure 1.**
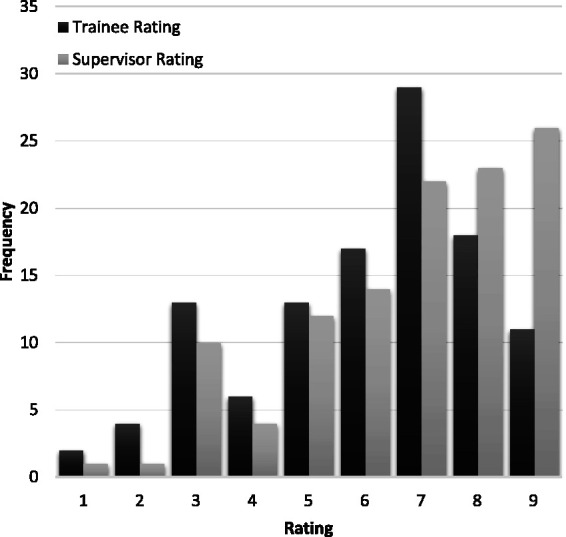
The frequency distribution of trainee and supervisor entrustment ratings.

**Figure 2.**
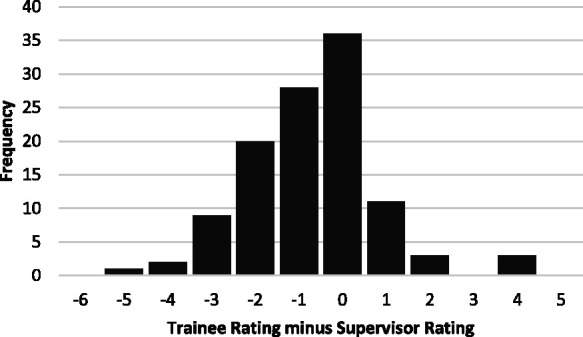
The frequency distribution of the difference between trainee and supervisor entrustment ratings, calculated by subtracting the supervisor rating from the trainee rating.

Figure 1 shows the frequency of entrustment ratings and Figure 2 shows the distribution of the difference between trainee and supervisor ratings (subtracting the supervisor rating from the trainee rating). Both trainees and supervisors use the full nine-point scale, with trainee ratings clustered around seven and supervisor ratings skewed towards nine. Trainee ratings were generally lower than supervisor ratings: the mean trainee rating was 6.1 (95% confidence interval (CI) 5.7 to 6.5), the mean supervisor rating was 6.8 (95% CI 6.4 to 7.1). Ratings agreed on 36 (32%) occasions, and 75 (66%) of trainee ratings were within one point of the supervisor rating. The difference between means was 0.7 (95% CI 0.4 to 1.0) and the two-tailed repeat measures t-test showed the difference was significant, *P* < 0.001. The correlation between trainee and supervisor ratings was *r* = 0.71 (95% CI 0.61 to 0.79) and *r*^2^ = 0.51. The degree of agreement between supervisor and trainee ratings measured by the ICC was 0.67 (95% CI 0.49 to 0.79), with no significant difference between recruitment sites. When analysed as a three-point scale based on the broad categories, the ICC was 0.58 (95% CI 0.45 to 0.69).

### Relationship of other variables to the difference in trainee and supervisor ratings

We found no significant correlation between the difference between trainee and supervisor ratings and any of the demographic variables collected (trainee gender, supervisor gender, trainee years of training, trainee training period, supervisor years of experience, times worked together, American Society of Anesthesiologists physical status score, case difficulty). We did find a significant negative correlation, *r* = −0.35 (95% CI −0.50 to −0.17) between the difference in ratings and supervisor rating, supporting a higher correlation in ratings as trainee expertise increased. When the supervisor rating was above the mean (i.e. 7, 8, or 9), six (8.45%) trainees rated themselves higher, 21 (29.6%) the same, and 44 (61.97%) rated themselves lower. When the supervisor rating was below the mean (i.e. 1 to 6), 11 (26%) trainees rated themselves higher, 15 (35.7%) the same, and 16 (38.1%) rated themselves lower. A higher-than-expected percentage of trainees with supervisor ratings below the mean rated themselves higher than their supervisors, and this result is statistically significant, χ^2^ (2, *N* = 113) = 8.67, *P* = 0.013.

### Comparison of trainee and supervisor comments

In general, allowing for differences in the detail recorded, most trainee-supervisor pairs with similar ratings reported similar reasons for the ratings they gave. To investigate further the potential explanation for the discrepancy between supervisor and trainee ratings, we chose to focus on the cases with greater disagreement between ratings. There were 12 cases in which the trainee rated themselves two or more points below the supervisor and six cases in which trainees rated themselves two or four points higher than their supervisors (Figure 2). There was a common theme observed in the cases in which trainees rated themselves lower, while we found two contributing explanations for the rating discrepancy when trainees rated themselves higher.

With the 12 cases in which trainees rated themselves lower, there was generally one critical aspect of the case when they needed guidance or assistance and hence rated themselves at the lower end of the scale. In these cases, the supervisor had not attached the same significance to this aspect, but rated the trainee’s performance higher based on the rest of their management. For example, in one case, the supervisor guided the trainee in performing a spinal anaesthetic using a paramedian approach, which they had not done before, after their initial attempts failed. The trainee judged this input crucial, rating their performance a two, whereas the supervisor remarked how minimal their input was and rated the case a seven.

The first issue in the cases in which trainees rated themselves substantially higher was that trainees don’t know what they don’t know. We will use one case to illustrate this point. The trainee reported they had appreciated the supervisor’s presence in case any difficulty occurred but had managed the case independently, rating seven on the entrustment scale. Conversely, from the supervisor’s point of view, they helped manage the case and rated the trainee three. The supervisor highlighted that the trainee did not consider how issues they had identified in their preoperative assessment related to one another. For example, the trainee identified that the patient had a high body mass index and was concerned with potential airway management difficulty. However, from the supervisor’s point of view, they did not consider that this would also make venous access difficult when they chose to use intravenous rather than inhalational anaesthesia. They also did not appreciate that difficult airway management and intravenous anaesthesia may both increase the risk of awareness and did not discuss this risk with the patient. The supervisor also reported needing to intervene, for example, to troubleshoot when the monitoring was not working. They noted the trainee was ‘prone to task fixation, e.g. waiting for relaxant to work but not attending to anaesthesia depth’. The supervisor commented that the trainee was ‘proficient at individual tasks but next step is to string tasks together, maintaining awareness of one parameter whilst managing another and anticipating the next job/task/problem’. The trainee appeared unaware of the further implications of their preoperative assessment and the supervisor’s intervention during the case.

The second issue we identified was a different perspective on whether a good outcome was sufficient for a high rating. Again, we will use one case to illustrate this point. The trainee rated their performance as seven, with the following comments conveying the tenor of their self-assessment: ‘Have done 8 (sub-Tenon’s blocks), no input required during insertion, but distant supervision still needed in case of troubleshoot … went well, developed rapport, patient cooperative.’

The supervisor noted ‘excellent block, technically sound’ but rated the performance a three. In their feedback, they noted the trainee should: ‘Increase confidence with technique, set up/ergonomics/efficiency and needs of anxious patient, developing an understanding of the anaesthetic requirements for different eye procedures … how to adapt when more technically difficult, ensure mention risk of permanent visual defect in consent.’

In this case, both the supervisor and trainee agreed that the case went well. The trainee rating indicates they felt the supervisor could be distant although aware of their anaesthesia plan. The supervisor rating reflects that they believe they need to be in the theatre suite and that the trainee might undertake some previously discussed actions if they were absent from the theatre. As the trainee managed the case and it proceeded uneventfully, the trainee may consider this the required level of supervision. We infer from the supervisor’s rating and comments that they have considered the trainee’s lack of experience in adapting to potential difficulties when deciding on their rating. Rather than only focusing on the outcome, they have also anticipated the trainee’s ability to adapt to difficulty and manage what might have happened when determining their rating.

## Discussion

In this study of 113 supervisor-trainee pairs across three sites, we found substantial concordance^27^ between trainees’ and supervisors’ ratings on the level of supervision required for a case using an entrustment scale. Trainee ratings were consistently lower than supervisor ratings, with a mean difference of 0.7. The absolute agreement of the trainee rating with the supervisor rating was 0.67, and the correlation was 0.71 with *r*^2^ = 0.51. Hence, the trainee rating explained 51% of the variance in the supervisor rating, which is considered a substantial degree of covariance^27,28^ in social science research where many factors may influence outcomes, and our results are comparable to desired levels of rater agreement in other studies.^24,29–33^ There was no significant correlation between demographic factors and the difference in ratings. The trainee rating better agreed with the supervisor rating as the supervisor rating rose. The principal reason for trainees rating themselves substantially lower than their supervisor was attending to a vital aspect of the case that the supervisor had not recognised. Trainees rated themselves substantially higher when they were unaware of aspects of their performance supervisors considered salient. Trainee ratings were also higher when supervisors reduced their rating to account for trainees’ perceived lack of readiness to deal with potential complications that had not occurred in the observed performance.

Our finding that the trainee rating better agreed with the supervisor rating when the supervisor rating was higher is also significant. While correlation does not imply causation, this enhanced concordance suggests that those performing better have more insight into how the supervisor will rate their performance. This is consistent with previous work showing self-assessment improves as competence improves,^25^ and augurs well for senior trainees becoming consultants who will manage their own learning. We did not find any significant correlation between demographic factors and the difference in ratings. While for this secondary analysis, our sample size only allowed the detection of a correlation greater than 0.25, smaller correlations are still seen as meaningful in behavioural research.^28^ Hence, while we can say that none of the demographic variables that we studied had a large effect on the difference in ratings, we cannot exclude a smaller yet still potentially interesting effect. The discussion of rater agreement has become more nuanced in recent years. Traditionally, the aim has been to minimise variation in performance assessment through training to improve assessor judgement and monitoring to ensure rigour and minimise assessor bias.^34^ As constructivist views on learning and assessment have risen to prominence, differences between assessor ratings have been re-interpreted as representing legitimately different yet valuable perspectives rather than unwanted variation or error.^34,35^ However, as new members in a community of practice, there is a need for trainees entering a profession to master its accepted practices and join its shared understanding of competence.^36^ Our results suggest that the entrustment scale provides a means to promote a shared understanding of competence between trainees and supervisors. If self-assessment is best thought of as a learning strategy, as we have described above, then the discussion between trainees and supervisors on the reasoning underlying their respective judgements is likely to be even more important than the actual ratings themselves.^37^

While we have not captured that discussion in this study, we have analysed the comments trainees and supervisors made when justifying their ratings. The principal reason for trainees rating themselves substantially lower than their supervisor was attending to what was, for them, a key aspect of the case that the supervisor had not recognised. In this instance, diverging views between supervisors and trainees on what is salient to rating performance highlight the value of personal reflection in self-assessment and learning from practice. Conversely, trainees rated themselves substantially higher when they were unaware of what supervisors considered were important aspects of the desired performance standard. The discrepancy in these cases may reflect trainee ignorance or ‘unknown unknowns’.^38^ Everyone makes errors of omission because we cannot know what we don’t know; however, the less expert we are, the more unknown unknowns there are.^25^ Trainees overestimating their ability to manage such cases safely is likely to be amenable to improvement based on supervisor feedback. In both these instances, discrepancies in ratings may stimulate useful discussion and reflection for the trainee, in which they can calibrate their assessment and judgement to improve their future practice.^39^

The other cause of discrepancy was when the supervisors’ rating reflected their judgement of the trainees’ ability to deal with potential but unobserved complications. The anticipatory nature of this judgement did not occur to the trainees in these cases. This may again reflect ‘unknown unknowns’;^38^ the discrepancy may simply reflect trainees’ ignorance of potential complications, leading them to overestimate their ability to manage future cases safely. Another possible cause of this discrepancy is whether the entrustment scale was interpreted as prospective or retrospective.^40^ In a retrospective scale, the level of supervision needed for the observed case determines the rating. In a prospective scale, the projected level of supervision required for a future case determines the rating.^40^ The ANZCA scale asks, ‘What level of supervision does the trainee require for THIS case overall?’. The emphasis on THIS may imply a retrospective judgement. The use of ‘does’, and the present tense in the more detailed scale descriptors in Tables 1 and 2, may imply a prospective judgement. This ambiguity may have resulted in supervisors taking a prospective view while trainees took a retrospective view, which could also explain these results. The way the entrustment scale is currently used within ANZCA WBAs could be ambiguous. Consistent prospective use would better reflect the purpose of entrustment, which is to use inferences from past performance to make a decision with future consequences.^40^

### Potential limitations

Our participants included supervisors and trainees from three institutions across two countries, which we think is a significant strength of our study. Fortunately, our inability to recruit our desired number of participants did not prevent us finding a significant result. Our study focused on anaesthesia; we cannot know how applicable our results are to other specialties with different work and assessment practices. There is some potential for selection bias to influence our results because our sample contained a higher proportion of advanced trainees and not all eligible trainees volunteered to participate. We also cannot exclude collusion between supervisor and trainee pairs and are reliant on our participants following the guidance to maintain independent ratings. The data we have collected only allow us to begin to explore the reasons underlying the differences in trainee self-assessment and supervisor assessment using an entrustment scale. Further research using complementary methods such as interviews could explore this topic in more depth.

## Conclusion

We found trainee self-assessment in WBA using an entrustment scale accorded substantially with supervisor assessment, suggesting the entrustment scale does provide a scaffold for a shared understanding of performance standards between trainees and supervisors. When ratings diverged, one party was ignorant of a vital aspect of performance or potentially interpreted the prospective nature of the scale differently. Based on our findings, such instances of divergent ratings provide a valuable opportunity for reflection and discussion. Training bodies could consider promoting discussion of divergent ratings to improve the feedback conversation and enhance trainee learning.
